# Negative Wealth Shock and Cognitive Decline and Dementia in Middle-Aged and Older US Adults

**DOI:** 10.1001/jamanetworkopen.2023.49258

**Published:** 2023-12-26

**Authors:** Liulu Pan, Bin Gao, Junpeng Zhu, Jing Guo

**Affiliations:** 1 Zhejiang University School of Medicine, Hangzhou, China; 2 Department of Psychiatry, The Second Affiliated Hospital, Zhejiang University School of Medicine, Hangzhou, China; 3 Center for Rehabilitation Medicine, Department of Psychiatry, Zhejiang Provincial People’s Hospital (Affiliated People’s Hospital, Hangzhou Medical College), Hangzhou, China; 4 Zhejiang Provincial Key Laboratory of Precision Diagnosis and Therapy for Major Gynecological Diseases, Women’s Hospital, Zhejiang University School of Medicine, Hangzhou, China

## Abstract

**Importance:**

As a financial hardship, negative wealth shock has been implicated in some adverse health outcomes. However, associations between negative wealth shock and cognitive decline and dementia have not been examined.

**Objective:**

To investigate whether negative wealth shock was associated with cognitive decline and incident dementia among middle-aged and older US adults.

**Design, Setting, and Participants:**

The Health and Retirement Study (HRS) is a prospective cohort study conducted biennially among US adults older than 50 years. Data from the HRS from calendar years 1996 to 2020 were analyzed from July 1 to 31, 2023. The final sample included 8082 participants with complete data of interest.

**Exposures:**

Wealth status was quantified with questionnaires. Negative wealth shock was defined as a loss of 75% or more in total wealth over a 2-year period. Asset poverty was defined as zero or less total net wealth.

**Main Outcomes and Measures:**

Cognitive function was assessed with the modified Telephone Interview for Cognitive Status (TICS-m). Dementia status was determined with TICS-m scores and proxy assessment.

**Results:**

Among 8082 participants included (mean [SD] age, 63.7 [5.7] years; 4179 women [51.7%] and 3903 men [48.3%]; 1111 Black [13.7%], 6689 White [82.7%], and 282 other [3.5%]), 1441 developed incident dementia over a median follow-up time of 14 (IQR, 7-20) years. Compared with participants who had positive wealth without shock, those with negative wealth shock had accelerated cognitive decline (β coefficient, −0.014 [95% CI, −0.027 to −0.001]; *P* = .03) and increased risks of dementia (hazard ratio [HR], 1.27 [95% CI, 1.11-1.46]; *P* < .001). Higher dementia risks were also found in participants with asset poverty at baseline (HR, 1.61 [95% CI, 1.30-2.00]; *P* < .001). Associations were found in White participants (HR, 1.34 [95% CI, 1.14-1.58]; *P* < .001) and participants younger than 65 years (HR, 1.38 [95% CI, 1.13-1.68]; *P* = .001) but not in other races and ethnicities or those 65 years or older.

**Conclusions and Relevance:**

In this cohort study, negative wealth shock was associated with accelerated cognitive decline and elevated risks of dementia among middle-aged and older US adults, with modifications by age and ethnicity. These findings should be confirmed by further prospective and interventional studies.

## Key Points

**Question:**

Is an experience of negative wealth shock—a loss of 75% or more in total wealth over a 2-year period—associated with cognitive decline and dementia risks among middle-aged and older US adults?

**Findings:**

In this cohort study of 8082 participants, those with negative wealth shock had faster decline in cognition and elevated risks of dementia when compared with those who had positive wealth without shock.

**Meaning:**

These findings suggest that negative wealth shock is a risk factor for cognitive decline and dementia in middle-aged and older adults.

## Teaser

This cohort study examines associations of negative wealth shock with cognitive decline and incident dementia in middle-aged and older adults in the US.

## Introduction

The worldwide number of cases with dementia was estimated at more than 50 million in 2019 and is expected to triple by 2050 worldwide.^1^ Dementia was regarded as the seventh leading cause of death in 2019^2^ and is expected to be the sixth leading cause of years of life lost in 2040, according to the globally forecasting data.^3^ Due to the absence of effective medical treatment, identification of early markers and populations at high risk for cognitive impairment and dementia are desirable for dementia prevention and research.

Lower socioeconomic status, including low income and low wealth, has been found to be associated with a later diagnosis^4^ and elevated risks of dementia.^5^ Negative wealth shock is defined as a sudden loss of wealth caused by rapid depletion of assets and accumulation of new debts,^6^ implying a decreased consumption of health-enhancing goods and services.^7^ Data from previous studies indicated that negative wealth shock induces physiological and psychological stress, such as higher blood pressure, inflammation,^8^ and mental disorders.^6^ Additionally, negative wealth shock has been reported to be associated with other health problems, including cardiovascular dysfunction,^9^ substance abuse,^10^ depression,^6^ and higher mortality.^11^ The aforementioned adverse outcomes (eg, inflammation, depression, and cardiovascular diseases) are also risk factors for incident dementia.^12^ A recent study has reported associations between negative wealth shock and cognitive function that was measured at only 1 time point.^13^ However, whether negative wealth shock is a risk factor for cognitive decline and dementia has not been evaluated previously. Based on the longitudinal data of the Health and Retirement Study (HRS),^14^ the aim of this study was to examine associations of negative wealth shock with cognitive decline and incident dementia in middle-aged and older adults in the US.

## Methods

### Study Design and Participants

The HRS, an ongoing longitudinal cohort study among US residents older than 50 years, was launched in 1992 and biennially collects information covering a wide range of topics, including demographic characteristics, lifestyle, health status, employment, and economic condition.^14^ The HRS had a high response rate of approximately 85% during follow-up.^14^ We used the RAND HRS Longitudinal File, which is a publicly available and streamlined data set containing the most commonly used HRS variables.^15^ The modified Telephone Interview for Cognitive Status (TICS-m) has been conducted to assess cognitive function since wave 3.^16^ In the present study, 24-year data from waves 3 (1996) to 15 (2020) were available for the data analysis. The HRS was approved by the University of Michigan Institutional Review Board. All participants provided written informed consent before interview. This study followed the Strengthening the Reporting of Observational Studies in Epidemiology (STROBE) reporting guideline.

The baseline waves corresponded to the first occurrence for participants with negative wealth shock and the first assessment on wealth status for participants without negative wealth shock during the whole study period. Among 13 651 participants, we excluded individuals who had no data during waves 3 to 15 (n = 256), were younger than 50 years (n = 1518), had prevalent dementia (n = 1823), had only 1 cognitive assessment during the study period (n = 1537), or had missing data on covariates (n = 435) (eFigure in Supplement 1). A total of 8082 participants with complete data of interest were retained in the data analysis.

### Assessment of Wealth Status

During the interview period, questionnaires were used to collect information on different wealth components, including housing, vehicles, businesses, checking and savings accounts, individual retirement accounts, stocks, and any other substantial assets. Outstanding debts including home mortgages, home loans, and other debts (eg, credit card balances, life insurance policy loans, loans from relatives, and medical debts) were also collected. The net value of total wealth was estimated as the sum of all wealth components less all debts and was adjusted to 2020 US dollars using the Consumer Price Index. Negative wealth shock was defined as a loss of 75% or more in total wealth between 2 consecutive interviews.^11^ According to a previous study,^11^ wealth levels were categorized into 3 exclusive groups of positive wealth without shock, asset poverty (zero or negative total net wealth) at baseline, and negative wealth shock.

### Assessment of Cognition and Dementia

We used a researcher-contributed data set that contains cognitive scores and dementia status and is publicly available on the HRS website.^17^ Cognitive performance was quantified with the TICS-m, which contains 3 tests consisting of immediate and delayed recall items, serial subtraction by 7, and counting backward. Total scores of TICS-m, ranging from 0 to 27 points, were calculated by summing the scores of each cognitive test, with higher scores indicating better cognitive function. Results from some previous studies have suggested that the TICS-m is validated for dementia screening.^16,18^ Cases of dementia were determined as those whose TICS-m scores are less than 6 points according to the criteria of Langa-Weir classification of cognitive function.^16,18^

Composite cognitive scores for proxy respondents were calculated with proxy assessment of limitations in 5 instrumental activities of daily living (taking medication, cooking, using the telephone, managing money, and shopping; range, 0-5 points), memory levels (excellent, very good, good, fair, and poor; range, 0-4 points), and the interviewer assessment of whether the respondents had cognitive impairment (no, maybe, and yes; range, 0-2 points). Total scores (range, 0-11 points) of proxy assessment were calculated, with higher scores indicating poorer cognitive performance. Participants were also classified as having dementia with proxy assessment scores of 6 or greater.^18^

### Covariates

Covariates were chosen based on associations with wealth status and cognitive function in the extant studies.^9,11,12^ Structured questionnaires were applied in the interview to collect information on age (in years), sex (male or female), self-reported race and ethnicity (Black, White, and other [includes American Indian or Alaska Native, Asian, Native Hawaiian or Other Pacific Islander, and any other self-specified race]; the latter category was combined to protect the privacy of the small number of participants), and years of education. Self-reported marital status was categorized into 3 levels: married or partnered; separated, divorced, or widowed; and never married. Smoking status was divided into 3 groups of never, current, and past smoking. Depressive disorders were defined as having scores on the 8-item short version of Center for Epidemiological Studies Depression Scale of 3 or greater.^19^ The multimorbidity burden (range, 0-8) was calculated as the total number of self-reported chronic health conditions, including hypertension, diabetes, cancer, lung diseases, heart diseases, stroke, psychological problems, and arthritis. Participants were asked whether they had engaged in vigorous physical activity 3 or more times per week. Disability was defined as having any difficulties in daily living activities of bathing, dressing, transferring, toileting, eating, and walking across a room. The total wealth at baseline was also used as a covariate in the association analysis.

### Statistical Analysis

Data were analyzed from July 1 to 31, 2023. The baseline characteristics of participants by wealth groups were compared using the analysis of variance for continuous variables and χ^2^ test for categorical variables. Associations between wealth status and longitudinal cognitive decline were estimated with mixed-effects linear regression models in which a participant-specific random intercept and slope of wealth status were applied. The mixed-effects linear regression models were constructed with wealth category, time, and a product term of wealth category × time as predictive factors and cognitive scores as the dependent variable. A significant interaction term between wealth category and time indicates differential rates of change in cognition as a function of wealth status. Positive wealth without shock defined the reference group. Model 1 was adjusted for age, sex, race and ethnicity, and years of education; model 2 was additionally adjusted for marital status, smoking, depressive disorder, comorbidity, physical activity, disability, and levels of total wealth (centralized transformation) at baseline.

Cox proportional hazards regression models were used to analyze associations between wealth categories and incident dementia. Ages at interview were used as the time scale. The survival time was calculated as years of follow-up between baseline and the occurrence of incident dementia, death, loss of follow-up, or the last visit (whichever came first). The product-term interactions between wealth status and each covariate were separately examined with the Wald test. The Cox proportional hazards regression models were adjusted for the same covariates as mixed-effects models. An example of statistical code for regression models is provided in eTable 1 in Supplement 1.

Some sensitivity analyses were performed for association analyses. First, participants who had follow-up time of less than 2 years were excluded. Second, the *APOE-E4* allele status was additionally adjusted among participants with complete data on *APOE-E4* measurement. Third, the cut point for negative wealth shock was set at 50%. Weighted Cox proportional hazards regression models were also conducted in consideration of the complex study design.

All data analyses were conducted with R, version 4.2.1 (R Project for Statistical Computing). Two-sided *P* < .05 was considered statistically significant.

## Results

### Baseline Characteristics

Among 8082 participants, the mean (SD) age was 63.7 (5.7) years. Men accounted for 3903 participants (48.3%) and women for 4179 (51.7%). A total of 1111 participants (13.7%) were Black, 6689 (82.7%) were White, and 282 (3.5%) were of other race or ethnicity. There were 1441 participants with no prevalent dementia at baseline who developed an incident dementia over a median follow-up time of 14 (IQR, 7-20) years, for a total of 107 356 person-years. There were 2185 participants who experienced negative wealth shock and 339 had asset poverty at baseline (Table 1). Compared with participants who had positive wealth without shock (n = 5558), individuals with negative wealth shock were more likely to be older, of Black race, current smokers, and separated, divorced, or widowed or never married. They were also more likely to have fewer years of education, depressive disorder, more comorbidities, less physical activity, disability, higher levels of total wealth, and poorer cognition at baseline. Differences in race and ethnicity, years of education, marital status, smoking, depressive disorder, comorbidity, physical activity, disability, total wealth, and cognitive scores were more apparent between the asset poverty group and the positive wealth without shock group. Compared with the included participants, excluded individuals were more likely to be younger, men, of Black race, married or partnered, and current smokers and to have disability and positive wealth without shock (eTable 2 in Supplement 1). Excluded participants also had less education, more comorbidities, lower levels of physical activity, and lower cognitive scores.

**Table 1.** Baseline Characteristics by Wealth Status

Characteristic	Participant group^a^				*P* value
	Total (N = 8082)	Positive wealth without shock (n = 5558)	Asset poverty at baseline (n = 339)	Negative wealth shock (n = 2185)	
Age, mean (SD), y	63.7 (5.7)	62.2 (4.4)	62.8 (4.7)	67.6 (6.8)	<.001
Sex					
Men	3903 (48.3)	2895 (52.1)	138 (40.7)	870 (39.8)	<.001
Women	4179 (51.7)	2663 (47.9)	201 (59.3)	1315 (60.2)	
Race and ethnicity					
Black	1111 (13.7)	524 (9.4)	119 (35.1)	468 (21.4)	<.001
White	6689 (82.7)	4890 (88.0)	195 (57.5)	1604 (73.4)	
Other^b^	282 (3.5)	144 (2.6)	25 (7.4)	113 (5.2)	
Educational attainment, mean (SD), y	12.41 (3.02)	12.76 (2.85)	10.74 (3.23)	11.76 (3.24)	<.001
Marital status					
Married or partnered	5909 (73.1)	4616 (83.1)	152 (44.8)	1141 (52.2)	<.001
Separated, divorced, or widowed	1947 (24.1)	825 (14.8)	172 (50.7)	950 (43.5)	
Never married	226 (2.8)	117 (2.1)	15 (4.4)	94 (4.3)	
Smoking					
Never	3043 (37.7)	2130 (38.3)	96 (28.3)	817 (37.4)	<.001
Past	3603 (44.6)	2517 (45.3)	139 (41.0)	947 (43.3)	
Current	1436 (17.8)	911 (16.4)	104 (30.7)	421 (19.3)	
Depressive disorder					
No	6364 (78.7)	4619 (83.1)	198 (58.4)	1547 (70.8)	<.001
Yes	1718 (21.3)	939 (16.9)	141 (41.6)	638 (29.2)	
No. of comorbidities, mean (SD)	1.61 (1.35)	1.35 (1.17)	2.19 (1.51)	2.17 (1.52)	<.001
Physical activity					
<3 Times/wk	4166 (51.5)	2644 (47.6)	231 (68.1)	1291 (59.1)	<.001
≥3 Times/wk	3916 (48.5)	2914 (52.4)	108 (31.9)	894 (40.9)	
Disability					
No	7085 (87.7)	5094 (91.7)	235 (69.3)	1756 (80.4)	<.001
Yes	997 (12.3)	464 (8.3)	104 (30.7)	429 (19.6)	
Total wealth as centralized transformation, mean (SD), $	−0.00 (1.04)	−0.02 (0.44)	−0.29 (0.04)	0.08 (1.88)	<.001
Cognitive scores, mean (SD)^c^	16.45 (4.17)	17.15 (3.99)	14.75 (4.42)	14.92 (4.12)	<.001
Dementia status					
No dementia	6641 (82.2)	4721 (84.9)	230 (67.8)	1690 (77.3)	<.001
Incident	1441 (17.8)	837 (15.1)	109 (32.2)	495 (22.7)	

^a^ Unless specified otherwise, data are expressed as No. (%) of participants. Percentages have been rounded and may not total 100.

^b^ Includes American Indian or Alaska Native, Asian, Native Hawaiian or Other Pacific Islander, or any other self-specified race.

^c^ Assessed using the modified Telephone Interview for Cognitive Status. Scores range from 0 to 27, with higher scores indicating better cognitive status.

### Associations Between Wealth Status and Cognitive Decline

Compared with participants who had positive wealth without shock, those with asset poverty at baseline (β coefficient, −0.619 [95% CI, −0.987 to −0.252]; *P* = .001) and negative wealth shock (β coefficient, −0.492 [95% CI, −0.669 to −0.315]; *P* < .001) had significantly lower cognitive scores at baseline (Table 2). In the analysis on longitudinal associations, participants with negative wealth shock (β coefficient, −0.014 [95% CI, −0.027 to −0.001]; *P* = .03) experienced faster decline in cognitive scores. The rate of change in cognitive scores was not significant (β coefficient, −0.024 [95% CI, −0.048 to 0.0001]; *P* = .05) for individuals in the asset poverty group.

**Table 2.** Associations Between Wealth Status and Cognitive Decline With Mixed-Effects Model

Wealth status	Model 1^a^		Model 2^b^	
	β Coefficient (95% CI)	*P* value	β Coefficient (95% CI)	*P* value
Baseline				
Positive wealth without shock	1 [Reference]	NA	1 [Reference]	NA
Asset poverty at baseline	−0.974 (−1.349 to −0.598)	<.001	−0.619 (−0.987 to −0.252)	.001
Negative wealth shock	−0.671 (−0.849 to −0.494)	<.001	−0.492 (−0.669 to −0.315)	<.001
Longitudinal				
Positive wealth without shock × time	1 [Reference]	NA	1 [Reference]	NA
Asset poverty at baseline × time	−0.026 (−0.051 to −0.002)	.03	−0.024 (−0.048 to 0.0001)	.05
Negative wealth shock × time	−0.015 (−0.028 to −0.003)	.02	−0.014 (−0.027 to −0.001)	.03

Abbreviation: NA, not applicable.

^a^ Adjusted for age, sex, race and ethnicity, and educational level.

^b^ Adjusted covariates in model 1 plus marital status, smoking, depressive disorder, number of comorbidities, physical activity, disability, and centralized total net wealth.

### Associations Between Wealth Status and Incident Dementia

**Figure 1.**
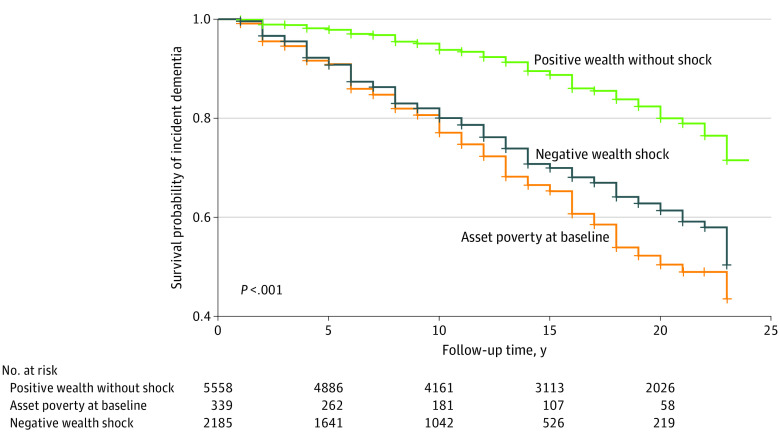
Incident Dementia by Wealth Status Kaplan-Meier curves show the survival probabilities of incident dementia by wealth status (log-rank test; *P* < .001).

The results of our Kaplan-Meier analysis suggest that the probabilities of incident dementia were different across wealth categories (Figure 1) (log-rank test; *P* < .001). The incidence rates of dementia were 10.20 (95% CI, 9.51-10.89) per 1000 person-years for positive wealth without shock, 29.33 (95% CI, 23.83-34.84) per 1000 person-years for asset poverty at baseline, and 22.97 (95% CI, 20.94-24.99) per 1000 person-years for negative wealth shock (Table 3). Compared with individuals who had positive wealth without shock, the hazard ratio (HR) for dementia was 1.61 (95% CI, 1.30-2.00; *P* < .001) for those with asset poverty at baseline and 1.27 (95% CI, 1.11-1.46; *P* < .001) for those with negative wealth shock.

**Table 3.** Associations Between Wealth Status and Incident Dementia With Cox Proportional Hazards Regression Model

Wealth status	No. of events/person-years	Incidence rate per 1000 person-years (95% CI)	Model 1^a^		Model 2^b^	
			HR (95% CI)	*P* value	HR (95% CI)	*P* value
Positive wealth without shock	837/82 088	10.20 (9.51-10.89)	1 [Reference]	NA	1 [Reference]	NA
Asset poverty at baseline	109/3716	29.33 (23.83-34.84)	2.11 (1.72-2.60)	<.001	1.61 (1.30-2.00)	<.001
Negative wealth shock	495/21 552	22.97 (20.94-24.99)	1.47 (1.29-1.67)	<.001	1.27 (1.11-1.46)	<.001

Abbreviation: HR, hazard ratio.

^a^ Adjusted for age, sex, race and ethnicity, and educational level.

^b^ Adjusted covariates in model 1 plus marital status, smoking, depressive disorder, number of comorbidities, physical activity, disability, and centralized total net wealth.

**Figure 2.**
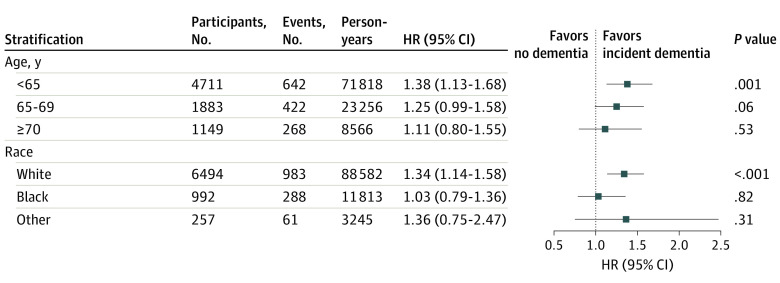
Associations Between Negative Wealth Shock and Dementia Stratified by Age and Ethnicity A fully adjusted Cox proportional hazards regression model was used to analyze associations between wealth status and incident dementia, with participants who had positive wealth without shock as the reference group. HR indicates hazard ratio.

We found significant interactions between wealth status and age (*P* = .01 for interaction) and but not between wealth status and race (*P* = .07 for interaction). Data analyses stratified by age groups (<65 years, ≥65 to <70 years, and ≥70 years) indicated that associations between negative wealth shock and dementia risks were positive among those younger than 65 years (HR, 1.38 [95% CI, 1.13-1.68]; *P* = .001) but not among the population 65 years or older (Figure 2). Positive associations were also found for White individuals (HR, 1.34 [95% CI, 1.14-1.58]; *P* < .001) but not for individuals of other races or ethnicities (Figure 2).

### Sensitivity Analysis

Similar results were found in the sensitivity analyses when removing participants with follow-up time of less than 2 years (n = 7987) (eTables 3 and 4 in Supplement 1), when additionally adjusting for *APOE-E4* allele status (n = 5452) (eTables 5 and 6 in Supplement 1), and when the cut point for the negative wealth shock was set at 50% (n = 7936) (eTables 7 and 8 in Supplement 1). Positive associations between negative wealth shock and dementia risks were also found in weighted Cox proportional hazards regression models (eTable 9 in Supplement 1).

## Discussion

In this prospective cohort study of 8082 adults older than 50 years living in the US, we found that negative wealth shock was associated with cognitive decline and elevated dementia risks, especially among White participants who were younger than 65 years. Our findings might shed light on the specific characteristics of populations who were at high risk of dementia and highlight the implementation of government policies and public health strategies to enhance the financial, social, and psychological supports for dementia prevention.

A wide range of studies^5,20,21^ have concluded that lower socioeconomic status in midlife, usually quantified by levels of income, education, and occupation, is associated with increased risks of dementia and dementia-related mortality. Similarly, our results showed associations between asset poverty and higher dementia risks. We also found that an experience of negative wealth shock was associated with accelerated decline in cognition and elevated risks of dementia among middle-aged and older adults, independent of covariates including levels of total net wealth at baseline.

In line with our findings, higher income volatility and more income drops of at least 25% over a 20-year period were associated with poorer cognitive function, smaller total brain volume, and worse microstructural integrity in the brain among 3287 adults with a mean age of 30 years.^22^ A 4-year follow-up study reported that associations between negative wealth shock and subsequent cognitive scores of older adults were significantly inverse in the US and China, but not in England or Mexico, suggesting the effects of macrolevel socioeconomic structures.^13^ Additionally, negative wealth shock could be regarded as a stressful life-course event. It has been reported that adverse events in late life, including the experience of major financial problems, were associated with increased risks of dementia among 12 789 community-dwelling Australians 70 years and older.^23^ The experimental evidence has suggested that psychological stress may increase the activation of the hypothalamic-pituitary-adrenal axis, inducing a dysregulation of glucocorticoid levels, which may increase brain vulnerability and pathological cognitive impairment.^24^

Data from 2 previous studies using HRS data indicated that wealth loss induced an impairment in mental health,^25^ and negative wealth shock was associated with short-term changes in depressive symptoms.^6^ Strong associations between early-, middle-, and late-life depression and subsequent incident dementia have been found in 1.4 million Danish citizens (median age, 50.8 [IQR, 34.7-70.7] years).^26^ Increased levels of systolic blood pressure and inflammatory biomarkers were found in 930 US older adults who experienced a wealth shock.^8^ Associations of cardiovascular problems and inflammation with impaired cognition and dementia have been documented.^12^ As a result, the pathway from negative wealth shock to dementia may also be linked by the intermediate conditions of depression, cardiovascular problems, and inflammation.

Our results suggest that associations between negative wealth shocks and incident dementia are stronger among participants younger than 65 years than among those 65 years or older. As people age, they are more likely to have an increase in positive emotions and a reduction in negative emotions and cope better with negative events.^27,28^ Older adults have higher levels of experience-based knowledge and lower levels of negative emotions, which are beneficial to financial decision-making.^28^ As a result, the adverse effect of negative wealth shock on dementia may be weaker in older participants than in younger ones.

In addition, significantly positive associations between negative wealth shock and dementia were found in the White but not in Black participants or those of other races or ethnicities. Some studies reported that White individuals have higher levels of depressive and anxiety symptoms compared with Black individuals.^29^ Black individuals are also more likely to have complete mental health than White individuals, implying a higher prevalence of common psychological disorders in White individuals.^30^ As a result, we suggest that White individuals appear to be more sensitive to such stressors as negative wealth shock and therefore have higher risks for psychological issues that are associated with dementia.

Nonsignificant associations in participants 65 years or older should be interpreted with caution, as the smaller sample size of those aged 65 to 69 years (n = 1883) and 70 years and older (n = 1149) in this study may have lower statistical power compared with the larger sample size of those younger than 65 years (n = 4711). Similarly, lack of association in Black participants or those of other races or ethnicities should also be noticed due to the smaller sample size compared with White participants.

Our findings linked the experience of financial hardship and dementia, which were useful to identify the vulnerable population and to make corresponding interventions. Some government policies including unemployment insurance^31^ and the earned income tax credit^32^ have been proposed to offset the burdens of wealth shock such as loss of a job and financial emergencies. The US Supplemental Nutritional Assistance Program has a substantial consumption-smoothing effect among low-income households during a period of income shock.^33^ Additionally, a greater cognitive resilience has been found among people with a better social support among US adults with a mean age of 63 years.^34^

### Limitations

This study has some limitations. First, the Langa-Weir classification of cognitive function has been reported to be validated for dementia screening among HRS participants 70 years or older^16^; however, whether it is appropriate for persons younger than 70 years has not been verified. Second, information on total wealth was collected via questionnaires that might lead to recall bias. Third, causes of negative wealth shock were not clarified, which precluded us from making strategies for the precise prevention. Fourth, there is a possibility of reverse causation in which the causes of negative wealth shock are associated with the risk of cognitive impairment, such as losing a job due to early cognitive decline or cognitive impairment in the context of serious illness. Fifth, participants 50 years and older were enrolled in the HRS, and the history of negative wealth shock before the enrollment could not be captured. Additionally, wealth changes after the occurrence of negative wealth shock were not considered, which might induce biased estimations.

## Conclusions

The findings of this cohort study suggest that an experience of negative wealth shock was associated with accelerated cognitive decline and elevated risks of dementia among the middle-aged and older US adults. The negative wealth shock–associated dementia risks were more apparent among White participants and those who were younger. Further prospective and interventional studies are warranted to confirm our findings.

## Notes

Supplement 1. **eFigure.** Flowchart of Participant Selection **eTable 1.** An Example Statistical Code for Regression Models **eTable 2.** Comparison of Characteristics Between the Included and Excluded Participants **eTable 3.** Associations Between Wealth Status and Cognitive Decline When Removing Participants With Follow-Up Time of Less Than 2 Years (n = 7987) **eTable 4.** Associations Between Wealth Status and Incident Dementia When Removing Participants With Follow-Up Time of Less Than 2 Years (n = 7987) **eTable 5.** Associations Between Wealth Status and Cognitive Decline When Additionally Adjusting for *APOE-E4* Allele Status (n = 5452) **eTable 6.** Associations Between Wealth Status and Incident Dementia When Additionally Adjusting for *APOE-E4* Allele Status (n = 5452) **eTable 7.** Associations Between Wealth Status and Cognitive Decline When the Cut Point for the Negative Wealth Shock Was Set at 50% (n = 7936) **eTable 8.** Associations Between Wealth Status and Incident Dementia When the Cut Point for the Negative Wealth Shock Was Set at 50% (n = 7936) **eTable 9.** Associations Between Wealth Status and Incident Dementia With Weighed Cox Regression Model (n = 8082)

Supplement 2.
Data Sharing Statement
