# Supplementary material for: Negative Wealth Shock and Cognitive Decline and Dementia in Middle-Aged and Older US Adults
Source: JAMA Netw Open. 2023 Dec 26;6(12):e2349258. doi: 10.1001/jamanetworkopen.2023.49258 (PMC10751595; doi:10.1001/jamanetworkopen.2023.49258)
Supplement: Supplement 1. — eFigure. Flowchart of Participant Selection eTable 1. An Example Statistical Code for Regression Models eTable 2. Comparison of Characteristics Between the Included and Excluded Participants eTable 3. Associations Between Wealth Status and Cognitive Decline When Removing Participants With Follow-Up Time of Less Than 2 Years (n = 7987) eTable 4. Associations Between Wealth Status and Incident Dementia When Removing Participants With Follow-Up Time of Less Than 2 Years (n = 7987) eTable 5. Associations Between Wealth Status and Cognitive Decline When Additionally Adjusting for APOE-E4 Allele Status (n = 5452) eTable 6. Associations Between Wealth Status and Incident Dementia When Additionally Adjusting for APOE-E4 Allele Status (n = 5452) eTable 7. Associations Between Wealth Status and Cognitive Decline When the Cut Point for the Negative Wealth Shock Was Set at 50% (n = 7936) eTable 8. Associations Between Wealth Status and Incident Dementia When the Cut Point for the Negative Wealth Shock Was Set at 50% (n = 7936) eTable 9. Associations Between Wealth Status and Incident Dementia With Weighed Cox Regression Model (n = 8082) [file jamanetwopen-e2349258-s001.pdf]

Supplementary Online Content

Pan L, Gao B, Zhu J, Guo J. Negative wealth shock and cognitive decline and dementia in middle-aged and older US adults. *JAMA Netw Open*. 2023;6(12):e2349258. doi:10.1001/jamanetworkopen.2023.49258

eFigure. Flowchart of Participant Selection
eTable 1. An Example Statistical Code for Regression Models
eTable 2. Comparison of Characteristics Between the Included and Excluded Participants
eTable 3. Associations Between Wealth Status and Cognitive Decline When Removing Participants With Follow-Up Time of Less Than 2 Years (n = 7987)
eTable 4. Associations Between Wealth Status and Incident Dementia When Removing Participants With Follow-Up Time of Less Than 2 Years (n = 7987)
eTable 5. Associations Between Wealth Status and Cognitive Decline When Additionally Adjusting for *APOE-ε4* Allele Status (n = 5452)
eTable 6. Associations Between Wealth Status and Incident Dementia When Additionally Adjusting for *APOE-ε4* Allele Status (n = 5452)
eTable 7. Associations Between Wealth Status and Cognitive Decline When the Cut Point for the Negative Wealth Shock Was Set at 50% (n = 7936)
eTable 8. Associations Between Wealth Status and Incident Dementia When the Cut Point for the Negative Wealth Shock Was Set at 50% (n = 7936)
eTable 9. Associations Between Wealth Status and Incident Dementia With Weighed Cox Regression Model (n = 8082)

This supplementary material has been provided by the authors to give readers additional information about their work.

© 2023 Pan L et al. *JAMA Network Open*.

eFigure. Flowchart of Participant Selection

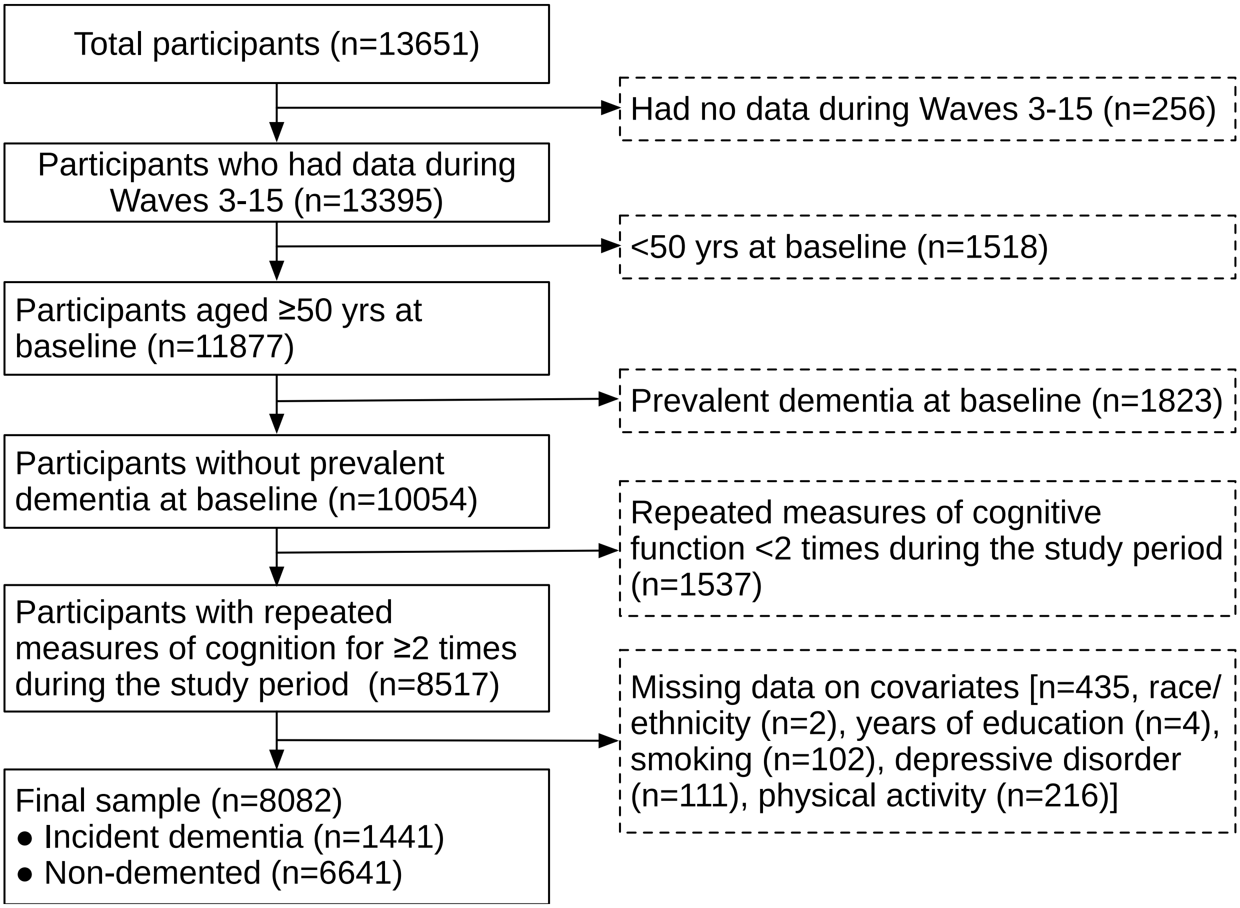

© 2023 Pan L et al. *JAMA Network Open*.

eTable 1. An Example Statistical Code for Regression Models

```
# ---------------------------------------------------- Labels of datasets and variables

# --- statistical software
# R version 4.2.1 (2022-06-23)

# --- data labels
# "data1", contains covariates, follow-up time, and dementia status. data1 is used for Cox regression models and weighted Cox regression models
# "data2", longitudinal data. data2 is used for mixed-effects linear regression models

# --- variable labels
# "hhidpn", identification number of participant
# "ftime", follow-up time
# "dem", dementia status
# "vtime", time duration between specific wave and the baseline wave
# "age_cov", age at baseline
# "sex_cov", sex at baseline
# "race_cov", race/ethnicity at baseline
# "edu_cov", years of education at baseline
# "mar_cov", marital status at baseline
# "smk_cov", smoking status at baseline
# "depress_cov", depressive disorder at baseline
# "comorb_cov", number of comorbidities at baseline
# "pa_cat_cov", physical activity at baseline
# "badl_dis_cov", disability at baseline
# "hatotb_adj0_center", total wealth at baseline
# "negshockcat", categories of wealth status
# "secu", cluster of study design
# "stratum", stratum of study design
# "wgtr", sampling weight of participant
```

```
# ---------------------------------------------------- Mixed-effects linear regression model
# Mixed-effects linear regression model
library("lmerTest")
library("lme4")

myfit<-
lmer(cog27~age_cov+sex_cov+race_cov+edu_cov+mar_cov+smk_cov+depress_cov+comorb_cov+pa_cat_cov+badl_dis_cov+hatotb_adj0_center+negshockcat*vtime+(1+negshockcat|hhidpn),data=data1)

summary(myfit)
```

© 2023 Pan L et al. *JAMA Network Open*.

```
# -------------------------------------------------- Cox regression model
library("survival")

myfit<-coxph(Surv(data1$ftime,data1$dem==1) ~
age_cov+sex_cov+race_cov+edu_cov+mar_cov+smk_cov+depress_cov+comorb_cov+pa_cat
_cov+badl_dis_cov+hatotb_adj0_center+negshockcat,data=data1)

summary(myfit)
```

```
# -------------------------------------------------- Weighted Cox regression model
# study design
library("survey")
sampdesign<-svydesign(id=~secu,
                strata=~stratum,
                weights=~wgtr,
                nest=TRUE,
                data=data2)

# weighted Cox regression model

myfit<-svycoxph(Surv(ftime,dem=='Incident') ~
age_cov+sex_cov+race_cov+edu_cov+mar_cov+smk_cov+depress_cov+comorb_cov+pa_cat
_cov+badl_dis_cov+hatotb_adj0_center+negshockcat,design=sampdesign)

summary(myfit)
```

© 2023 Pan L et al. *JAMA Network Open*.

eTable 2. Comparison of Characteristics Between the Included and Excluded Participants

Characteristics	Total [a]	Excluded	Included	*P* value
Sample size	10054	1972	8082	
Age (years), mean (SD)	63.33 (5.78)	61.64 (5.84)	63.69 (5.71)	< .001
Sex, n (%)				< .001
Male	5018 (49.91%)	1115 (56.54%)	3903 (48.29%)	
Female	5036 (50.09%)	857 (43.46%)	4179 (51.71%)	
Race/ethnicity, n (%)				< .001
White/Caucasian	8196 (81.60%)	1507 (76.81%)	6689 (82.76%)	
Black/African American	1468 (14.62%)	357 (18.20%)	1111 (13.75%)	
Other	380 (3.78%)	98 (4.99%)	282 (3.49%)	
Educational attainment (years), mean (SD)	12.32 (3.07)	11.94 (3.24)	12.41 (3.02)	< .001
Marital status, n (%)				< .001
Married/partnered	7500 (74.60%)	1591 (80.68%)	5909 (73.11%)	
Separated/divorced/widowed	2290 (22.78%)	343 (17.39%)	1947 (24.09%)	
Never married	264 (2.63%)	38 (1.93%)	226 (2.80%)	
Smoking, n (%)				< .001
Never	3617 (36.43%)	574 (31.09%)	3043 (37.65%)	
Past	4376 (44.08%)	773 (41.87%)	3603 (44.58%)	
Current	1935 (19.49%)	499 (27.03%)	1436 (17.77%)	
Depressive disorder, n (%)				.41
No	7728 (78.58%)	1364 (77.85%)	6364 (78.74%)	
Yes	2106 (21.42%)	388 (22.15%)	1718 (21.26%)	
Number of comorbidities, mean (SD)	1.64 (1.36)	1.77 (1.41)	1.61 (1.35)	< .001
Physical activity, n (%)				< .01
< 3 times/wk	5086 (52.21%)	920 (55.42%)	4166 (51.55%)	
≥ 3 times/wk	4656 (47.79%)	740 (44.58%)	3916 (48.45%)	
Disability, n (%)				.03
No	8773 (87.31%)	1688 (85.86%)	7085 (87.66%)	
Yes	1275 (12.69%)	278 (14.14%)	997 (12.34%)	
Cognitive scores, mean (SD)	16.32 (4.18)	15.74 (4.17)	16.45 (4.17)	< .001
Patterns of wealth change, n (%)				< .001
Positive Wealth Without Shock	757 (60.17%)	5558 (68.77%)	6315 (67.61%)	
Asset Poverty at Baseline	58 (4.61%)	339 (4.19%)	397 (4.25%)	
Negative Wealth Shock	443 (35.21%)	2185 (27.04%)	2628 (28.14%)	

Abbreviations: BMI, body mass index; SD, standard deviation; %, percentage.

[a] Characteristics were compared among 10054 participants who had data during Waves 3-15, aged ≥ 50 years, and had no prevalent dementia at baseline.

© 2023 Pan L et al. *JAMA Network Open*.

eTable 3. Associations Between Wealth Status and Cognitive Decline When Removing Participants With Follow-Up Time of Less Than 2 Years (n = 7987)

	Model 1 [a]		Model 2 [b]	
Wealth status	β (95% CI)	*P* value	β (95% CI)	*P* value
Baseline				
Positive wealth without shock	Reference		Reference	
Asset poverty at baseline	-0.941 (-1.288, -0.593)	< .001	-0.592 (-0.937, -0.248)	.001
Negative wealth shock	-0.678 (-0.850, -0.505)	< .001	-0.498 (-0.671, -0.326)	< .001
Longitudinal				
Positive wealth without shock × time	Reference		Reference	
Asset poverty at baseline × time	-0.027 (-0.051, -0.002)	.03	-0.024 (-0.048, -0.0002)	.05
Negative wealth shock × time	-0.014 (-0.027, -0.001)	.04	-0.013 (-0.025, 0.0003)	.06

Abbreviations: CI, confidence interval; β, coefficient.

[a] Model 1 was adjusted for age, sex, race/ethnicity, and education.

[b] Model 2 was adjusted covariates in Model 1 plus marital status, smoking, depressive disorder, number of comorbidities, physical activity, disability, and centralized total net wealth.

© 2023 Pan L et al. *JAMA Network Open*.

eTable 4. Associations Between Wealth Status and Incident Dementia When Removing Participants With Follow-Up Time of Less Than 2 Years (n = 7987)

Wealth status	Events/ person-years	Incidence rate per 1000 person-years (95% CI)	Model 1 [a]		Model 2 [b]	
			HR (95% CI)	*P* value	HR (95% CI)	*P* value
Positive wealth without shock	830/82034	10.12 (9.43, 10.81)	Reference		Reference	
Asset poverty at baseline	106/3708	28.59 (23.14, 34.03)	2.09 (1.69, 2.58)	< .001	1.59 (1.28, 1.99)	< .001
Negative wealth shock	486/21519	22.58 (20.58, 24.59)	1.48 (1.30, 1.68)	< .001	1.28 (1.12, 1.47)	.001

Abbreviations: CI, confidence interval; HR, hazard ratio.

[a] Model 1 was adjusted for age, sex, race/ethnicity, and education.

[b] Model 2 was adjusted covariates in Model 1 plus marital status, smoking, depressive disorder, number of comorbidities, physical activity, disability, and centralized total net wealth.

© 2023 Pan L et al. *JAMA Network Open*.

eTable 5. Associations Between Wealth Status and Cognitive Decline When Additionally Adjusting for *APOE-ε4* Allele Status (n = 5452)

	Model 1 [a]		Model 2 [b]	
Wealth status	β (95% CI)	*P* value	β (95% CI)	*P* value
Baseline				
Positive wealth without shock	Reference		Reference	
Asset poverty at baseline	-0.975 (-1.320, -0.631)	< .001	-0.760 (-1.189, -0.330)	.001
Negative wealth shock	-0.672 (-0.844, -0.500)	< .001	-0.525 (-0.727, -0.323)	< .001
Longitudinal				
Positive wealth without shock × time	Reference		Reference	
Asset poverty at baseline × time	-0.025 (-0.049, -0.001)	.04	-0.014 (-0.041, 0.013)	.31
Negative wealth shock × time	-0.014 (-0.027, -0.001)	.03	-0.012 (-0.026, 0.002)	.09

Abbreviations: CI, confidence interval; β, coefficient.

[a] Model 1 was adjusted for age, sex, race/ethnicity, and education.

[b] Model 2 was adjusted covariates in Model 1 plus marital status, smoking, depressive disorder, number of comorbidities, physical activity, disability, centralized total net wealth, and *APOE*-ε4 allele status (dichotomous).

© 2023 Pan L et al. *JAMA Network Open*.

eTable 6. Associations Between Wealth Status and Incident Dementia When Additionally Adjusting for *APOE-ε4* Allele Status (n = 5452)

Wealth status	Events/ person-years	Incidence rate per 1000 person-years (95% CI)	Model 1 [a]		Model 2 [b]	
			HR (95% CI)	*P* value	HR (95% CI)	*P* value
Positive wealth without shock	574/65048	8.82 (8.10, 9.55)	Reference		Reference	
Asset poverty at baseline	72/2660	27.07 (20.82, 33.32)	2.32 (1.80, 2.99)	< .001	1.78 (1.37, 2.32)	< .001
Negative wealth shock	350/16955	20.64 (18.48, 22.81)	1.59 (1.36, 1.85)	< .001	1.40 (1.19, 1.64)	< .001

Abbreviations: CI, confidence interval; *APOE*-ε4, apolipoprotein E genotype ε4; HR, hazard ratio.

[a] Model 1 was adjusted for age, sex, race/ethnicity, and education.

[b] Model 2 was adjusted covariates in Model 1 plus marital status, smoking, depressive disorder, number of comorbidities, physical activity, disability, centralized total net wealth, and *APOE*-ε4 allele status (dichotomous).

© 2023 Pan L et al. *JAMA Network Open*.

eTable 7. Associations Between Wealth Status and Cognitive Decline When the Cut Point for the Negative Wealth Shock Was Set at 50% (n = 7936)

	Model 1 [a]		Model 2 [b]	
Wealth status	β (95% CI)	*P* value	β (95% CI)	*P* value
Baseline				
Positive wealth without shock	Reference		Reference	
Asset poverty at baseline	-1.020 (-1.404, -0.637)	< .001	-0.689 (-1.064, -0.314)	< .001
Negative wealth shock	-0.472 (-0.625, -0.319)	< .001	-0.386 (-0.538, -0.234)	< .001
Longitudinal				
Positive wealth without shock × time	Reference		Reference	
Asset poverty at baseline × time	-0.032 (-0.056, -0.007)	.01	-0.030 (-0.054, -0.005)	.02
Negative wealth shock × time	-0.018 (-0.029, -0.008)	.001	-0.017 (-0.027, -0.007)	.001

Abbreviations: CI, confidence interval; β, coefficient.

[a] Model 1 was adjusted for age, sex, race/ethnicity, and education.

[b] Model 2 was adjusted covariates in Model 1 plus marital status, smoking, depressive disorder, number of comorbidities, physical activity, disability, centralized total net wealth, and *APOE*-ε4 allele status (dichotomous).

© 2023 Pan L et al. *JAMA Network Open*.

eTable 8. Associations Between Wealth Status and Incident Dementia When the Cut Point for the Negative Wealth Shock Was Set at 50% (n = 7936)

Wealth status	Events/ person-years	Incidence rate per 1000 person-years (95% CI)	Model 1 [a]		Model 2 [b]	
			HR (95% CI)	*P* value	HR (95% CI)	*P* value
Positive wealth without shock	443/47152	9.40 (8.52, 10.27)	Reference		Reference	
Asset poverty at baseline	109/3711	29.37 (23.86, 34.89)	2.29 (1.84, 2.85)	< .001	1.83 (1.46, 2.30)	< .001
Negative wealth shock	879/45390	19.37 (18.09, 20.65)	1.43 (1.26, 1.63)	< .001	1.34 (1.18, 1.53)	< .001

Abbreviations: CI, confidence interval; *APOE*-ε4, apolipoprotein E genotype ε4; HR, hazard ratio.

[a] Model 1 was adjusted for age, sex, race/ethnicity, and education.

[b] Model 2 was adjusted covariates in Model 1 plus marital status, smoking, depressive disorder, number of comorbidities, physical activity, disability, centralized total net wealth, and *APOE*-ε4 allele status (dichotomous).

© 2023 Pan L et al. *JAMA Network Open*.

eTable 9. Associations Between Wealth Status and Incident Dementia With Weighed Cox Regression Model (n = 8082)

Wealth status	Events/ person-years	Incidence rate per 1000 person-years (95% CI)	Model 1 [a]		Model 2 [b]	
			HR (95% CI)	*P* value	HR (95% CI)	*P* value
Positive wealth without shock	837/82088	10.20 (9.51, 10.89)	Reference		Reference	
Asset poverty at baseline	109/3716	29.33 (23.83, 34.84)	2.16 (1.73, 2.70)	< .001	1.64 (1.31, 2.06)	< .001
Negative wealth shock	495/21552	22.97 (20.94, 24.99)	1.54 (1.31, 1.81)	< .001	1.34 (1.15, 1.57)	< .001

Abbreviations: CI, confidence interval; HR, hazard ratio.

[a] Model 1 was adjusted for age, sex, race/ethnicity, and education.

[b] Model 2 was adjusted covariates in Model 1 plus marital status, smoking, depressive disorder, number of comorbidities, physical activity, disability, and centralized total net wealth.

© 2023 Pan L et al. *JAMA Network Open*.
