# Supplementary material for: Negative Wealth Shock and Cognitive Decline and Dementia in Middle-Aged and Older US Adults
Source: JAMA Netw Open. 2023 Dec 26;6(12):e2349258. doi: 10.1001/jamanetworkopen.2023.49258 (PMC10751595; doi:10.1001/jamanetworkopen.2023.49258)
Supplement: Supplement 2. — Data Sharing Statement [file jamanetwopen-e2349258-s002.pdf]

Data Sharing Statement

Pan. Negative Wealth Shock and Cognitive Decline and Dementia in Middle-Aged and Older US Adults. *JAMA Netw Open*. Published December 26, 2023.
doi:10.1001/jamanetworkopen.2023.49258

Data
Data available: No

Additional Information
Explanation for why data not available: Data used in this study are publicly available on the website of HRS (https://hrs.isr.umich.edu/about).
